# Social Networks as a Tool for Evidence-Based Health Education: Umbrella Review

**DOI:** 10.3390/nursrep14030168

**Published:** 2024-09-06

**Authors:** Teresa Sufrate-Sorzano, Olatz Corton-Carrasco, María-Elena Garrote-Cámara, Noelia Navas-Echazarreta, Pablo del Pozo-Herce, Marco Di Nitto, Raúl Juárez-Vela, Iván Santolalla-Arnedo

**Affiliations:** 1 Research Group in Care and Health, Department of Nursing, University of La Rioja, 26006 Logroño, Spain; maria-elena.garrote@unirioja.es (M.-E.G.-C.); noelia.navas@unirioja.es (N.N.-E.); pablo.pozo@quironsalud.es (P.d.P.-H.); raul.juarez@unirioja.es (R.J.-V.); ivsantol@unirioja.es (I.S.-A.); 2 Cruces University Hospital, Paediatric Emergency Service, 48903 Barakaldo, Spain; olatz.cortoncarrasco@osakidetza.eus; 3 Unie University, 28040 Madrid, Spain; 4 Department of Health Sciences, University of Genoa, 16132 Genoa, Italy; marco.dinitto@unige.it

**Keywords:** health education, health care professionals, nursing, social media, social network

## Abstract

Background: The number of people who access social networking sites continues to increase at an exponential rate. The use of technology is an essential skill for nursing professionals and its development represents a challenge in improving health education, promotion and care. The objective of this systematic review is to analyse the use of social networking sites by healthcare professionals as an intervention tool for evidence-based public health education. Methods: The protocol of this umbrella review was registered in the International Prospective Register of Systematic Reviews (CRD42023407249). Searches were carried out in MEDLINE (PubMed), Web of Science, CINAHL, PsycINFO (EBSCOhost), and Cochrane Library of Systematic Reviews, in February 2023. A narrative synthesis of the results was conducted. Results: 1896 articles were found, of which 15 reviews fulfilled the inclusion criteria. Social networks broadened the profession; they were YouTube, X (formerly Twitter), Facebook, and Instagram. The target population was mainly young professionals, and they came across different topic areas that addressed health education. Conclusions: It is important to have information backed by scientific evidence to make health decisions. Health professionals active on social networking sites have a unique opportunity to educate the public about health by sharing scientific evidence in an accessible and clear way, which helps to combat misinformation.

## 1. Introduction

The Internet has brought about a revolutionary change in society, transforming how the population communicates and impacting immediate accessibility in the social, cultural, and educational spheres [1]. Along these lines, social networks make it possible to disseminate knowledge and facilitate interaction among society. This allows for a faster and broader exchange of ideas, interdisciplinary collaboration, and active community participation in public health issues, which can improve the implementation of educational interventions and their impact on society [2].

From this technological breakthrough, so-called “social communities” emerged, allowing users to engage in social interaction with large and small audiences synchronously or asynchronously [3]. The first social networking that facilitated interaction between users dates back to 1997, after this moment, a substantial evolution in the area continued in 2002 with the creation of LinkedIn, Myspace in 2003, Facebook in 2004, YouTube in 2005, Twitter in 2006, Instagram and Pinterest in 2010, and currently the most popular application, created in 2016, TikTok [4,5].

Social networking can be classified into horizontal (users are not defined by a specific topic or activity) and vertical (grouping users under a specific topic) [6].

The Internet and, specifically, social networking sites, are fundamental tools for the dissemination of information, but at the same time, the source of unlimited and immediate information can be considered a disadvantage [7]. Although social media is used as a source of information, according to the latest 2023 Digital News Report, only 40% of the United States surveyed trust the information contained [8].

The social media, content creators, called ‘influencers’, have the ability to influence users’ opinions and behaviours, which is relevant for population health education [9,10]. The use of social media in health brings together a group of individuals and health professionals with common health-related interests, sharing content and interacting with each other and the population [11]. To this end, professionals especially nurses have to develop in contexts mediated by digital platforms that optimise the health experiences of patients and the general population [12].

### Health Promotion and Education in Times of E-Professionalism

Health promotion is considered “the global political and social process that encompasses actions aimed at modifying the social, environmental and economic conditions to favour a positive impact for individual and collective health”, by the World Health Organization [13]. If health promotion is understood as an educational process of learning to autonomously control the population’s health through the resources available to individuals, the fundamental role of health education in health promotion is clear [14].

Health education is defined as the discipline in charge of guiding and organising educational processes to positively influence health knowledge, practices, and customs of individuals and communities, with one of the objectives being the participation of the population in their self-care practices [15]. Among the barriers that hinder the health education process are the difficulty in accessing health services, access to evidence-based information on health, and the difficulty in controlling chronic diseases [16]. For this reason, health education has been considered one of the most significant projects regarding the attempt to fight for equity and consolidate a population capable of making suitable health decisions [17].

Providing and administering care to the population is one of the main functions of nursing professionals. In this case, it involves health education and training the population in self-management and management of their health and illness, thereby playing a very important role in the community’s state of health, and this work is one of the most representative functions of the nursing profession [18].

In 2009, the term “e-professionalism” was defined for the first time as “the attitudes and behaviours that reflect paradigms traditional forms of professionalism that are manifested through digital media”. Through this concept, the need to evolve at the pace of society is clarified by responding appropriately, since the forms of inter- and intrapersonal communication are modified in relation to the evolution of social networking sites [19].

Digital media have led to a collaborative educational venture in addition to the dissemination of relevant content. Many professionals in their field disseminate health knowledge through social networking, blogs, and other platforms, with nursing being one of the professions experiencing a sharp increase [20].

Nursing plays a crucial role in health education, particularly through social networking, serving as a bridge between scientific knowledge and the general public. Nursing professionals can use these platforms to disseminate verified information, combat misinformation, and promote healthy habits. Moreover, their presence on social networks allows for direct interaction with the community, facilitating access to personalized health advice, and fostering a culture of prevention. This digital education strategy contributes significantly to improving health literacy and reducing knowledge gaps across diverse populations.

Social networking sites have become powerful tools for communication and information exchange, connecting millions of people worldwide. However, the relevance and authenticity of the information shared on these platforms is a critical and complex issue. Although social networks enable the rapid dissemination of news and opinions, they are also fertile ground for the spread of disinformation and fake news. The immediacy and global reach of these platforms can amplify inaccurate or manipulated content, underscoring the importance of fostering a culture of verification and critical thinking among users. To ensure that the information we consume and share is reliable, it is essential to use reputable sources and verify data before dissemination. This is the only way to harness the potential of social networking in a constructive and responsible manner.

Today, the application of technology is a fundamental part of the work of nursing professionals, with major advances available to them. These advances present a challenge in how to effectively integrate them into education and care. For this reason, the research team posed the following research question: Are social networks used by health professionals as intervention tools in evidence-based health education? The objective of this systematic review was to analyse the use of social networks by health professionals as an intervention tool in evidence-based health education.

## 2. Materials and Methods

We began with a first pilot search in PubMed to find out the systematic reviews that met the established inclusion criteria. Different reviews were obtained that fit the criteria and this led to an umbrella review being carried out, following the Joanna Briggs Institute (JBI) methodology [21]. JBI’s systematic approach is aimed at providing a comprehensive and objective synthesis using rigorous and transparent methods. The research protocol is registered in the International Prospective Register of Systematic Reviews (PROSPERO) with registration code CRD42023407249 and the report was written according to the Preferred Reporting Items for Overviews of Reviews (PRIOR) statement [22] as can be seen in Supplementary File S1.

### 2.1. Search Strategy

A systematic search was conducted for systematic reviews published from 2001 to the 24th and 26th of February 2023. The search strategy was updated on the 2nd of September 2024. The search was first performed in PubMed, applying Mesh terms, and free text terms, and using wildcards if deemed appropriate. Then, the final search was tailored for use in all other databases considered: MEDLINE (PubMed), CINAHL, PsycINFO (via EBSCOhost), Web of Science, and Cochrane Library of Systematic Reviews. One author (Author 6) with experience in systematic reviews conducted the literature search. The search terms that guided the search were “social media”, “social network”, “social networking”, “health education”, “health literacy”, “health personnel”, and “systematic review”. Supplementary File S2 shows the complete search strategy.

### 2.2. Inclusion and Exclusion Criteria

We included all systematic reviews written in English, Spanish, or Italian related to health education for the population through social networking sites. Primary studies, books, book sections, and grey literature were excluded, as well as systematic reviews that did not focus on our topic.

### 2.3. Outcome Measures

The primary outcome of this study was to identify the improvement of health literacy in the general population made through social networking sites. Considering the aim of this review, we defined health literacy as the knowledge, practices, and habits of individuals and communities concerning their health.

### 2.4. Review Selection

All reviews were retrieved from each database and were uploaded to Rayaan^®^ and duplicates were removed [23]. Rayaan^®^ (Developed by Qatar Computing Research Institute in 2016), is a tool designed to assist in conducting systematic reviews. It facilitates the process of selecting, organizing, and analysing articles, allowing for collaborative work in reviewing large volumes of publications. In one of the reviews, the full text was not available on any platform, so we contacted the corresponding author directly by mail, but we did not receive a response and so it was not included. According to the eligibility criteria, two authors independently (Author 1 and Author 2) reviewed the titles and summaries. Subsequently, they assessed the appropriateness of the review texts that could be included. Due to the independence of the reviewers throughout the process, any discrepancies were resolved by discussion. When consensus was not reached, it was resolved through consultation with a third reviewer (Author 3).

### 2.5. Quality Assessment

Two authors (Author 1 and Author 2) analysed the methodological quality of the selected reviews, including a new reviewer (Author 3) in the analysis for decisions that did not have consensus. The Critical Appraisal Skills Program Spanish (CASPe) tool was used for systematic reviews [24]. This tool for assessing a systematic review is designed to assess quality by providing a framework to identify bias, assess methodological rigour, and determine the relevance of studies. It is made up of ten items and considers three areas. Are the results valid? What are the results? Will the results help locally? The researchers considered that, if there was at least a response “no” or “unclear” in one of the ten items, it would infer moderate quality. If there were at least three, it would be defined as low quality. The quality level obtained with the analysis was considered both in the description of results and in its generalisation.

### 2.6. Data Extraction

After the initial screening, data were collected and extracted to answer the research question (‘Are social networks used by health professionals as intervention tools in evidence-based health education?’) using a collection form incorporating characteristics of systematic reviews such as reference and year, overall objective, type of review, databases, period covered, and results. As in all other phases, this was performed independently by two researchers (Author 1 and Author 2) with the inclusion of a third researcher (Author 3) to arbitrarily resolve conflicts.

### 2.7. Data Synthesis

The Joanna Briggs Institute’s methodology manual for umbrella reviews recommends that the results of an umbrella review be reported to provide syntheses on existing research relevant to a particular topic [21]. Data from the included systematic reviews were summarised in narrative form, and results were presented both in table form and within the text.

## 3. Results

**Figure 1:**
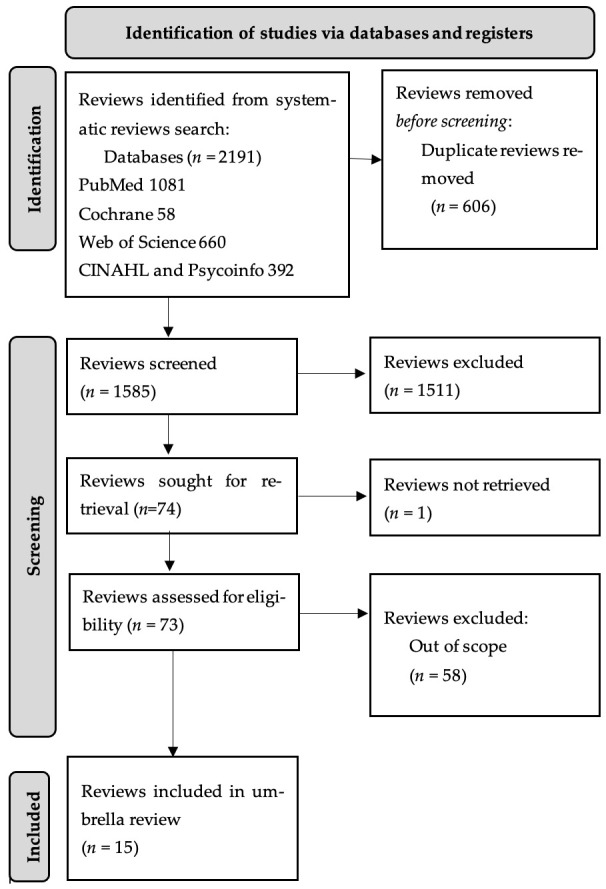
PRIOR flow diagram.

The search yielded a total of 2191 articles (PubMed 1.081, CINAHL and PsycINFO 392, Web of Science 660, and Cochrane, 58). Initially, we started by eliminating the 606 duplicates, and reducing the number to 1585 articles, which were then reviewed based on title and summary to assess their relevance and eligibility criteria, resulting in the exclusion of 1511 records. Subsequently, 74 articles were examined, and after excluding 58 documents that did not address our research question, and 1 due to lack of access to text or response from the corresponding author, a total of 15 reviews that aligned with our objective of analysing the use of social networking as an intervention tool for public health education were systematically analysed. None of the included reviews included a meta-analysis. The procedure is described in Figure 1. Supplementary File S3 shows the revisions eliminated in the screening phase. 

### 3.1. Evaluation of Methodological Quality

In the quality evaluation methodology, seven reviews were given a high-quality level [25,26,27,28,29,30,31] and eight reviews were deemed as moderate quality [32,33,34,35,36,37,38,39]. All were included in the review since their results can be generalised and applied to the population. The main findings are presented as a synthesis narrative. The results obtained are shown in Table 1.

### 3.2. Characteristics of the Included Studies

The publication date of the included systematic reviews spans from 2013 to 2022, and the time range used by their authors for the search ranged from the start of the database containing the data to the year 2022. The reviews they analysed studied primary or direct educational video content on YouTube (Table 2).

The 15 articles selected in this systematic review were categorised into 4 articles [25,28,38,39] that analysed a total of 510 videos and, therefore, 11 articles [26,27,29,30,31,32,33,34,35,36,37] in which 270 studies were included as primaries. Of these studies, descriptive observational design was the most frequent (*n*= 88), followed by qualitative investigations (*n* = 73), controlled randomised trials (*n* = 49), quasi-experimental studies (*n* = 27), analytical observational studies (*n* = 15), cross-sectional studies (*n* = 16), and experimental studies (*n* = 2).

We primarily mapped the studies addressed in systematic reviews that were relevant to the objectives of this study and assessed the overlap between all reviews. No overlap between primary studies was found and no review overlapped with another. Two primary studies were cited twice in the fifteen reviews considered, which yielded an overall corrected area deck (CCA) of 0.08. This finding is perceived as advantageous for research since it indicates that the reviews considered did not duplicate the analysis of the primary studies.

A summary of the general characteristics of the included reviews is reported in Table 3.

### 3.3. Summary of Evidence

Results are presented in sub-sections including the social networking sites subsequently used to deliver health education, the target population of health education on social media, and the subject areas addressed by social media for health education, in order to provide a proper analysis and facilitate understanding of the results obtained.

### 3.4. Social Networking Sites Most Used for Health Education

The social networking sites for health education most cited in the selected reviews are, firstly, YouTube [25,26,27,28,30,31,32,33,34,36,38,39] followed by X (Formerly Twitter, [26,27,31,32,33,34,35], Facebook [26,27,30,31,33,34,37], and Instagram [26,27,34]. Other tools analysed for this purpose with less reference were Myspace [31,34], Reddit [26,27], Wiki [29,31], Flickr [34], LinkedIn [26], Tumblr [34], and WeChat [27,34]. Some specific research refers to the use of informative platforms such as discussion forums [26,34,35], support groups [26,35], or blogs [30,31,34]. Among the reviews included, eleven analyse several social networking sites while four articles reference a unique one [25,28,38,39].

It is worth mentioning that numerous reviews, most of which are of moderate quality, highlight the role of the health professional in social media health education as one of the most active content creators [26,28,32,33]. However, in the review on education for heparin administration, more than 49% of the videos were made by non-health professionals [25].

#### 3.4.1. YouTube

It is the most commonly used medium for the dissemination of health information. Specifically, five of the articles analyse the content of this platform about health education [25,28,38,39], reporting moderate and high-quality analysis levels. However, some research highlights the lack of quality information in the published videos [25,26,36,38,39]. It should be noted that the location of the videos within the platform is in YouTube’s “education” section [28,39].

Specifically, the work by Wittenberg states that more than half of the videos did not provide evidence-based information; 49% of the videos were made by unqualified people; and more than 70% of the videos did not cite bibliographical references (Wittenberg et al.). Likewise, the review by Gun et al. [25] on heparin administration education and that of Gupta et al. [38] about peripheral neuropathy stated that the information in almost half of the selected videos was erroneous.

According to recent research by Ulep et al., YouTube could be used for education by 2022, as videos on this platform posted by healthcare professionals acquired significantly higher quality criteria than those made by non-professionals [26].

#### 3.4.2. X (Formerly Twitter)

X is analysed in eight of the reviews as one of the tools further used for health education [26,27,31,32,33,34,35,36]. Specifically, the research by Goodyear et al., with a high-level quality score, addressed the promotion of physical activity and healthy nutrition through “tweets”, gamification, and the creation of groups to answer questions, observing changes positive in the behaviours [27]. Following suit, a 2022 review concludes that, although the information on the platform regarding hearing loss and tinnitus was not properly examined, 1% misinformation was found among the tweets studied [26].

While it is true, and specifically when analysing this network for the prevention of breast cancer, there was confusion, especially among X users, due to the false information disseminated on the subject on this network [33].

#### 3.4.3. Facebook

Among the interventions studied on Facebook for health education are the creation of groups [26,27,37] and gamification [27,37]. Facebook is used as a platform where the anonymity of the user can be used to receive information through private groups, private text messaging systems, or private access pages, which is considered a significant advantage, for example, in addressing sexual health education and promotion [34]. However, the dissemination of misinformation, false information or disinformation is higher than that disseminated on YouTube and X according to the research by Ulep et al., which is rated with a high level of quality. [26]

Odone et al.‘s work shows that in a survey conducted to assess the willingness to be vaccinated against influenza, people who accessed the Facebook page where the vaccination promotion campaign was carried out with informative content, videos, and promotional games, were almost 2.5 times more willing to be vaccinated than those who did not receive any intervention [30].

#### 3.4.4. Instagram

The Instagram social networking is only analysed in three of the articles selected from those that range in quality levels from moderate to high [26,27,34]. Ulep et al. (2022) reveal that the weekly use of this network is the highest, along with Facebook [26]. However, this is not one of the sources with a high percentage of users seeking health information [26,34].

#### 3.4.5. Social Networks Specific, Forums, Groups, and Blogs

Three studies were found to indicate the existence and use of social networks to address specific issues [29,31,37]. In their work, Ridout et al. cite three social media dedicated to mental health education, Horyzons, Rebound, and Mindmax, which are based on interaction with and between users, gamification, and online spaces for evidence-based group problem solving [37].

In 2018, DeAngelis et al. presented a wide range of social networks for various chronic patient groups, including websites to educate on disease self-management skills and interactive platforms for information dissemination [29]. The third review only mentions the name of the social networks, without analysing the target population or objectives [31].

Discussion forums, support groups, and blogs were the other social media analysed [26,30,31,34,35], and it is noted that blogs that contradict health interventions, such as blogs against vaccination campaigns, could have a negative impact on the population [30]. 

### 3.5. Target Population of Health Education in Social Networking Sites

**Figure 2:**
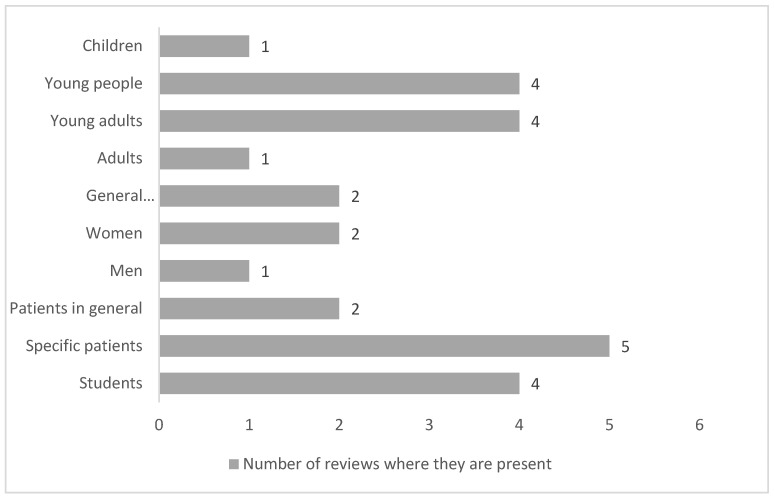
The target population was found in the reviews analysed.

Figure 2 shows the ratio of the number of reviews that address the different population groups.

Only four of the articles reviewed did not contain specific information on the target audience of the interventions [26,28,38]. Age was dependent on the objective of the research. For example, in the case of the promotion of vaccination campaigns, the target age was adults over 65 years old, and in the promotion of mental and sexual health, the target audience was young people. As such, the students are set up as the main target population age group of health education [27,30,34,37].

In terms of the gender specificity of the population, there are only two reviews, one focusing on breast cancer screening in women and one paper that discusses sexual health promotion with male- and female-only interventions [33,34]. Similarly, they have identified articles without specifying groups of people where the target population is described as the general population [31,34].

Regarding group population, they have registered reviews including women as target population primigravida [27], caregivers [39], patients with chronic diseases, mental health disorders [37], cancer [27,35,39], and obesity [27].

Educational interventions aimed at health professionals for training purposes were identified, although this is not the focus of this study [31].

### 3.6. Thematic Areas Addressed by Social Networking Sites Media for Health Education

A great heterogeneity was observed among the subject areas of health education in social networking sites (Table 4).

The results obtained can be grouped into three over-arching topics: patients with specific illnesses [26,28,29,32,33,35,36,38,39], promotion of healthy habits [27,30,31,34,37], and teaching techniques [25].

Under the classification of education for patients with specific illnesses, some have pointed out interventions on education and pain management [28,39], chronic disease self-management [29,31], sexual health [31], breast cancer screening [33], and heart failure education [32], bladder cancer [35], peripheral neuropathy [38], hearing impairments [26], and kidney stones [36]. In these, they discuss different aspects, such as symptomatology [31,38], diagnosis [31], treatment and pharmacological self-administration [26,31,38], online support [26,27,29,31,32,34], information dissemination [26,27,29,31,34], behaviour changes [34], awareness [32,33], and causes and complications [38].

In the promotion of lifestyle habits, they have found themes focused on the promotion of physical activity and nutrition [27], giving up tobacco [31], the promotion of mental health [37], and sexual health [34]. In terms of teaching techniques, only one review reference focuses on patient training for heparin self-injection [25].

## 4. Discussion

An umbrella review is crucial because it synthesises and evaluates evidence from multiple systematic reviews on a topic, providing a comprehensive overview. This methodology allows for the identification of patterns, discrepancies, and gaps in existing research, which facilitates informed and robust decision making. It also provides a hierarchy of the quality of the available evidence, improving the validity and applicability of conclusions for clinical practice or public policy [40,41]. This umbrella review of the use of social media in the dissemination of evidence-based health education by health professionals is critical to understanding the impact and effectiveness of these platforms in health promotion.

This is the first umbrella review that explores the use of social networking sites as an intervention tool for health education. The quality assessment revealed a moderate-to-high level of quality.

Social networks are growing in popularity year after year until they reach a high percentage of users, and with it, the emergence of new influencers. Social networks are a powerful tool that, in addition to offering collaboration between users and disseminators, means a new dimension for health and specifically, for health education. Among the results found, some authors recommend resorting to platforms and social networking where the material has been developed by healthcare professionals, but in some cases, there may be difficulties in verifying this authorship.

Some research shows the favourable results that were obtained with the interventions analysed in health education through social networks, with only the work of Goodyear et al. being evaluated with high quality [27,32,36,37]. While most of the results were optimistic, Goodyear claims that several results are negative, showing YouTube to be an unreliable source of health-related information [27]. In the same vein, the 2022 research found commercial health education videos on the YouTube platform where the influencer remained neutral or addressed the advantages and disadvantages of different health interventions in only 32% of the videos analysed [42].

Currently, misinformation, over-information, or the dissemination of information by unqualified people and without scientific evidence are features that generate mistrust among users [26]. Consequently, this has a negative impact on the implementation and promotion of social networking sites as a tool in the health sector.

The platforms used for health education are mainly YouTube, Twitter, Facebook, and Instagram. Surprisingly, the result of this review does not match the social network with the fastest growth at the moment, TikTok, which has not been mentioned in any of the narratives [43]. Therefore, the opportunity to simultaneously use different social networks to promote health education actions, as well as research that analyses the use of this network, should be allowed.

In terms of the target population of health education interventions, it is noted that the target audience for most interventions is young people and students, specifically in terms of mental and sexual health promotion [31,34,37]. However, the articles reviewed do show a perceived lack of data and precision in terms of which population the interventions were targeted at.

The main subject areas addressed in health education on social networking sites were found to be information on relevant topics, such as breast cancer screening, on platforms such as Facebook and YouTube [33].

The results obtained show a lack of specific training for carers and family members, who are key individuals in supporting the patient and are responsible for providing most of the care and attention required by the patient [42].

## 5. Limitations

The quality of the umbrella review depends on the quality of the included studies and in our review, 53% of the included studies show a moderate level of quality. It is also not possible to know whether all published articles report on the same thing, since they have different objectives.

More studies could be found by modifying the search procedure, which is a limitation of this research. For example, ‘social network’ should not be a search term because the result only includes reviews on social networks, which means that it has already been used. However, the authors believe that the conclusions would be largely the same.

## 6. Implications for Nursing Practice, Research, and Education

This review represents a before and after in the perspective of the use of education for health in social networks. Disinformation and dissemination of information lacking scientific basis pose a risk to public health. This highlights the need for more quality studies to improve communication, in the short and long term. With the topics covered in this review, the analysis of the population, the interventions and platforms used, and the subject areas addressed in greater depth, it provided valuable information for future health education interventions on social networking sites.

Finally, the work of health professionals specifically nursing professionals, was found to be strongly related to health education; thus, it could be considered to introduce social media management as a tool for educating the population. The results of this review suggest that it would be highly interesting to introduce education on the use of social media in terms of health by nurses, as well as to include this person as an active creator of content with scientific evidence on social media.

## 7. Conclusions

Health education interventions through social media are now a reality, but the need for information and outreach activity to be supported by scientific evidence is evident. Health professionals are seen as one of the most important content creators on social networks. In this context, professionals could carry out activities to educate the population on how to search for valid and scientifically backed information. It is important to have information backed by scientific evidence to make health decisions. Health professionals active on social media have a unique opportunity to educate the public about health by sharing scientific evidence in an accessible and clear way, which helps to combat misinformation.

For future research, it is recommended that studies provide more details on the determination of evidence-based information obtained from social networking sites. When searching the Internet for health information for teaching, it is essential that the sites visited have valid and reliable peer-reviewed journals.

Educational videos on techniques and self-management, as well as explanatory blogs on physical, mental, or sexual health education with a scientific basis, could be widely used and are more effective tools for health education. The population is far from being fully educated on social networks, so nursing plays an important role in providing users with lower percentages of misinformation and quality information. 

Part of the role of nursing is to empower patients to take an active role in their own care. By educating patients about the use of social networks, nurses can help patients develop self-care and self-efficacy skills. Nurses, within the multidisciplinary team in which they perform their work, are well suited to provide education to patients about social networking because of their proximity, availability, and the educational nature of their role. This combination of factors allows nurses to address this issue in an effective and meaningful way. Finally, including communication experts to work with healthcare teams may be the most effective way.

## Figures and Tables

**Table 1:** Methodological quality analysis tool for systematic reviews—CASPe. Each criterion is scored as Yes, No, or Unclear. Chronological order.

Authors															
Items	Moorhead et al. 2013 [31]	Wit-ten-berg et al. 2014 [39]	Odone et al. 2015 [30]	Gupta et al. 2016 [38]	DeAngelis et al. 2018 [29]	Jamnadass et al. 2018 [36]	Ridout et al. 2018 [37]	Heathcote et al. 2019 [28]	Tariq et al. 2019 [35]	Dobrossy et al. 2020 [33]	Martin et al. 2020 [34]	Eliya et al. 2021 [32]	Goodyear et al. 2021 [27]	Gun et al. 2022 [25]	Ulep et al. 2022 [26]
1	Y	Y	Y	Y	Y	Y	Y	Y	Y	Y	Y	Y	Y	Y	Y
2	Y	Y	Y	Y	Y	Y	Y	Y	Y	Y	Y	Y	Y	Y	Y
3	Y	Y	Y	Y	Y	Y	Y	Y	N	N	N	Y	Y	Y	Y
4	Y	U	Y	U	Y	U	U	Y	U	U	U	U	Y	Y	Y
5	Y	Y	Y	Y	Y	Y	Y	Y	Y	Y	Y	Y	Y	Y	Y
6	Y	Y	Y	Y	Y	Y	Y	Y	Y	Y	Y	Y	Y	Y	Y
7	N.S.	N.S.	N.S.	N.S.	N.S.	N.S.	N.S.	N.S.	N.S.	N.S.	N.S.	N.S.	N.S.	N.S.	N.S.
8	Y	Y	Y	Y	Y	Y	Y	Y	Y	Y	Y	Y	Y	Y	Y
9	Y	Y	Y	Y	Y	Y	Y	Y	Y	Y	Y	Y	Y	Y	Y
10	Y	Y	Y	Y	Y	Y	Y	Y	Y	Y	Y	Y	Y	Y	Y

Each criterion is scored Y: Yes. N: No. U: Unclear. N.S.: Narrative Synthesis. Item 1: Did the review address a focused question? Item 2: Did the authors look for the right types of papers? Item 3: Do you think all the important, relevant studies were included? Item 4: Did the review authors do enough to assess the quality of the included studies? Item 5: If the results of the review have been combined, was it reasonable to do so? Item 6: What are the overall results of the review? Item 7: How precise are the results? Item 8: Can the results be applied to the local population? Item 9: Were all important outcomes considered? Item 10: Are the benefits worth the harm and costs?

**Table 2:** The number of investigations included in reviews is systematic.

Author	Research
Moorhead et al., 2013 [31]	98 studies
Odone et al., 2015 [30]	19 studies
Wittenberg et al., 2014 [39]	43 videos
Gupta 2016 [38]	200 videos
De Angelis et al., 2018 [29]	7 studies
Heathcote et al., 2019 [28]	106 videos
Jamnadass et al., 2018 [36]	10 studies
Ridout et al., 2018 [37]	9 studies
Tariq et al., 2019 [35]	15 studies
Dobrossy et al., 2020 [33]	17 studies
Martin et al., 2020 [34]	60 studies
Eliya et al., 2021 [32]	3 studies
Goodyear et al., 2021 [27]	16 studies
Gun et al., 2022 [25]	161 videos
Ulep et al., 2022 [26]	16 studies

**Table 3:** Data extraction form with the characteristics of the 15 systematic reviews included. Chronological order.

Reference and Years	General Target	Review Typology	Databases and/or Platforms	Period Covered	Main Findings
Moorhead et al., 2013 [31]	Identify the uses, benefits, and limitations of social media for health communication between the public, patients, and healthcare professionals. Identify current gaps to offer recommendations about health communication.	Systematic review	CSA Illumina, Cochrane Library, Communication Abstracts, EBSCOhost CINAHL Complete, Embase, ISI Web of Knowledge, Medline, PsycINFO, PubMed Central, Web of Science.	From inception until 3 April 2013	The use of social networking sites for health communication offers a number of advantages: increased accessibility to health information, social/emotional support, public health surveillance and the possibility to influence health policy. The quality and reliability of information need to be reviewed.
Wittenberg et al., 2014 [39]	Explore the availability of cancer pain management videos and instructions on YouTube and determine the extent to which these videos address the role of caregivers in cancer pain management.	Systematic review	YouTube.	From inception until 2 April 2014	79% of videos were created by non-professional users, 49% did not provide the qualifications of the creator, and 70% did not cite the sources of information. More than 90% showed the sources of financing. The videos about skill development are not considered solid.
Odone et al., 2015 [30]	Gather available systematic evidence on the effectiveness of interventions that apply new means to promote vaccination and increase vaccination coverage.	Systematic review	Embase, Medline.	From inception until 1 November 2014	Text messaging, access to vaccination campaign websites, use of patient web portals and computerised reminders increase vaccination coverage rates. While there is great potential for vaccine coverage through social media, the available data are sparse and more rigorous research is needed.
Gupta et al., 2016 [38]	Review the systematic information on YouTube on peripheral neuropathy.	Systematic review	YouTube.	From inception until 16 January 2015	Half of the videos were not evidence-based so you must be cautious when using YouTube videos as resources for patients. Directing the patient to a video on YouTube created by professionals can save time in consultations, motivate them to ask, and educate them about their disease.
Angelis et al., 2018 [29]	Summarise the evidence related to the use of social media by healthcare professionals to facilitate chronic disease self-management.	Systematic review	Cochrane Central Register of Controlled Trials, CINAHL, Embase, ERIC, Medline, PsycINFO.	From inception until 21 March 2018	Discussion forums and collaborative projects appear to be promising resources for healthcare professionals to help patients with illness self-management.
Jamnadass et al., 2018 [36]	Determine whether social media and search engines play a role in the management and/or prevention of kidney stones.	Systematic review	CINAHL, Cochrane Library, Embase, Embase Classic, +Embase, Medline, PubMed, Scopus.	From inception until 26 June 2018	Social networks and search engines provide valuable information to patients with kidney stones. However, although the information provided about aspects of diet was good, it was not complete enough to include tips about other aspects related to kidney stone prevention.
Ridout et al., 2018 [37]	Identify available systematic evidence on the use of social network-based interventions to support mental health in young people up to the age of 25, assess their effectiveness, appropriateness and safety, and identify gaps and opportunities for future research.	Systematic review	PsycINFO, PubMed.	From inception until 18 December 2018	Evidence suggests that young people find social network-based interventions very helpful, engaging and supportive. Future studies need to address the lack of high-quality evidence on their effectiveness in reducing mental health symptoms.
Heathcote et al., 2019 [28]	Browse availability characteristics and content of the YouTube videos that address the neuroscience of pain.	Systematic review	YouTube.	From inception until 11 February 2019	YouTube contains various videos that professionals, patients, and families can view to access information on the neuroscience of pain. It remains to be determined to what extent patients are able to learn information, to what extent the videos promote behaviour change and to what extent they can be useful for practice clinics.
Tariq et al., 2019 [35]	Assess the use of the Internet and social media by people with bladder cancer and their carers. Synthesise the quality of the online resources for patients with bladder cancer.	Systematic review	Embase, PsycINFO, PubMed, Web of Science, Scopus.	From inception until 23 April 2019	The review highlights that bladder cancer, despite its high prevalence worldwide, remains under-represented in evidence-gathering on patients’ information needs and the potential role of online spaces.
Dobrossy et al., 2020 [33]	Assess the volume, participants and content of breast screening on social media. Find out whether screening organisers can use social media as a health education channel for patients.	Systematic review	EBSCO, PubMed, ScienceDirect, Springer, Web of Science.	From inception until 15 April 2020	Websites dedicated to breast screening that ensure the quality of information and provide a space for question-and-answer forums are useful for sharing and exchanging experiences.
Martin et al., 2020 [34]	Describes existing studies on participatory online intervention methods used to promote sexual health among adolescents and young adults.	Systematic review	Aurore database of Institut National d’Études Demographiques, PubMed.	From inception until 31 July 2020	Specific online interventions for young people’s sexual health have demonstrated their feasibility, practical interest and attractiveness, but their effectiveness has not yet been sufficiently evaluated.
Eliya et al., 2021 [32]	Evaluate the source profile and content of posts on X (formerly Twitter) and YouTube about heart failure.	Systematic review	Embase, Medline, PubMed, Twitter, YouTube.	From inception until 21 November 2019	YouTube is the platform for the dissemination of cardiac failure knowledge, with contributions from institutions, healthcare professionals and patients. The target population of both networks is professionals and, less frequently, patient education.
Goodyear et al., 2021 [27]	Update on social media interventions for physical activity, physical activity and dietetics. Analyse features of interventions that lead to changes in behaviour related to physical activity and diet. Evaluate the differences in results in different population groups.	Systematic review	Embase, EBSCO Education, Medline, Wiley, and Scopus.	From inception until 5 June 2021	Social media interventions can positively modify behaviours related to physical activity and diet. They have provided new insights into the uses to which responsible policy makers, practitioners, organisations and researchers can put them.
Gun et al., 2022 [25]	Examine the content, reliability, popularity and quality of YouTube videos for self-monitoring of subcutaneous low-molecular-weight heparin.	Systematic review	YouTube.	From August 2021 to April 2022	Healthcare professionals should ensure the accuracy and quality of specific videos on self-administration of low molecular weight heparin injections before recommending YouTube to patients. Policies are needed to limit the spread of health misinformation by evaluating the evidence of information on social media sites such as YouTube.
Ulep et al., 2022 [26]	Synthesise the research related to the use of social media related to social issues in connection with hearing loss, tinnitus, and disorders vestibular.	Systematic review	Academic Search Complete, CINAHL, Psychology and Behavioural Sciences Collection, PubMed (including Medline).	From inception until 2022	Online discussions about hearing and vestibular disorders are evident, although inconsistencies in the studies’ procedures make comparison difficult. Misinformation is a problem that must be addressed in clinical consultations and through other public health media.

**Table 4:** Areas of health education topics addressed in the systematic reviews.

Subject Area	Author
Health promotion and/or education	Moorhead et al., 2013 [31]
Physical activity and nutrition	Goodyear et al., 2021 [27]
Mental health	Moorhead et al., 2013 [31]
	Ridout et al., 2018 [37]
Sexual health	Moorhead et al., 2013 [31]
	Martin et al., 2020 [34]
Improving vaccine acceptance and coverage	Odone et al., 2015 [30]
Encouragement to give up tobacco	Moorhead et al., 2013 [31]
Education about pain	Heathcote et al., 2019 [28]
	Wittenberg et al., 2014 [39]
Information dissemination about kidney stones	Jamnadass et al., 2018 [36]
Information divulgation about cancer	Wittenberg et al., 2014 [39]
	Dobrossy et al., 2020 [33]
	Tariq et al., 2019 [35]
Education about tinnitus, loss of hearing, and vestibular disorders	Ulep et al., 2022 [26]
Education about peripheral neuropathy	Gupta et al., 2016 [38]
Education on the Heparin administration	Gun et al., 2022 [25]

## Data Availability

All data generated or analysed during this study are included in this published article [and its Supplementary Information files].

## Supplementary Materials

The following supporting information can be downloaded at: https://www.mdpi.com/article/10.3390/nursrep14030168/s1, Supplementary File S1: PRIOR Checklist; Supplementary File S2: Search strategy in an electronic database; Supplementary File S3: Excluded reviews.

## Public Involvement Statement

No public or patient contribution.

## Guidelines and Standards Statement

This manuscript was drafted against the Preferred Reporting Items for Overviews of Reviews.
