# Supplementary material for: Social Networks as a Tool for Evidence-Based Health Education: Umbrella Review
Source: Nurs Rep. 2024 Sep 6;14(3):2266–82. doi: 10.3390/nursrep14030168 (PMC11417878; doi:10.3390/nursrep14030168)
Supplement: Supplementary file 1 [file nursrep-14-00168-s001.zip › nursrep-3112200 Supplementary.pdf]

Supplementary File S1: PRIOR Checklist

(Gates M, Gates A, Pieper D, et al. Reporting guideline for overviews of reviews of healthcare interventions: development of the PRIOR statement. ***BMJ*** **2022;378:e070849. doi:10.1136/bmj-2022-070849.)**

**Section** Topic	**#**	**Item**	**Location reported**
**TITLE**			
Title	1	Identify the report as an overview of reviews.	Page 1
**ABSTRACT**			
Abstract	2	Provide a comprehensive and accurate summary of the purpose, methods, and results of the overview of reviews.	Page 1
**INTRODUCTION**			
Rationale	3	Describe the rationale for conducting the overview of reviews in the context of existing knowledge.	Pages 1-3
Objectives	4	Provide an explicit statement of the objective(s) or question(s) addressed by the overview of reviews.	Page 3
**METHODS**			
Eligibility criteria	5a	Specify the inclusion and exclusion criteria for the overview of reviews. If supplemental primary studies were included, this should be stated, with a rationale.	Page 3
	5b	Specify the definition of 'systematic review' as used in the inclusion criteria for the overview of reviews.	Page 3
Information sources	6	Specify all databases, registers, websites, organizations, reference lists, and other sources searched or consulted to identify systematic reviews and supplemental primary studies (if included). Specify the date when each source was last searched or consulted.	Page 3
Search strategy	7	Present the full search strategies for all databases, registers and websites, such that they could be reproduced. Describe any search filters and limits applied.	Suppl. 1
Selection process	8a	Describe the methods used to decide whether a systematic review or supplemental primary study (if included) met the inclusion criteria of the overview of reviews.	Page 4
	8b	Describe how overlap in the populations, interventions, comparators, and/or outcomes of systematic reviews was identified and managed during study selection.	Page 3-4
Data collection process	9a	Describe the methods used to collect data from reports.	Page 5
	9b	If applicable, describe the methods used to identify and manage primary study overlap at the level of the comparison and outcome during data collection. For each outcome, specify the method used to illustrate and/or quantify the degree of primary study overlap across systematic reviews.	Page 4
	9c	If applicable, specify the methods used to manage discrepant data across systematic reviews during data collection.	Page 5
Data items	10	List and define all variables and outcomes for which data were sought. Describe any assumptions made and/or measures taken to identify and clarify missing or unclear information.	Page 3
Risk of bias assessment	11a	Describe the methods used to *assess* risk of bias or methodological quality of the included systematic reviews.	Page 4
	11b	Describe the methods used to *collect* data on (from the systematic reviews) and/or *assess* the risk of bias of the primary studies included in the systematic reviews. Provide a justification for instances where flawed, incomplete, or missing assessments are identified but not re-assessed.	Page 4
	11c	Describe the methods used to *assess* the risk of bias of supplemental primary studies (if included).	Page 4
Synthesis methods	12a	Describe the methods used to summarize or synthesize results and provide a rationale for the choice(s).	Page 4
	12b	Describe any methods used to explore possible causes of heterogeneity among results.	Page 4
	12c	Describe any sensitivity analyses conducted to assess the robustness of the synthesized results.	Page 4
Reporting bias assessment	13	Describe the methods used to *collect* data on (from the systematic reviews) and/or *assess* the risk of bias due to missing results in a summary or synthesis (arising from reporting biases at the levels of the systematic reviews, primary studies, and supplemental primary studies, if included).	Page 4
Certainty assessment	14	Describe the methods used to *collect* data on (from the systematic reviews) and/or *assess* certainty (or confidence) in the body of evidence for an outcome.	Page 4
**RESULTS**			
Systematic review and supplemental primary study selection	15a	Describe the results of the search and selection process, including the number of records screened, assessed for eligibility, and included in the overview of reviews, ideally with a flow diagram.	Page 4-5
	15b	Provide a list of studies that might appear to meet the inclusion criteria, but were excluded, with the main reason for exclusion.	Suppl. 2

**Section** Topic	**#**	**Item**	**Location reported**
Characteristics of systematic reviews and supplemental primary studies	16	Cite each included systematic review and supplemental primary study (if included) and present its characteristics.	Table 2
Primary study overlap	17	Describe the extent of primary study overlap across the included systematic reviews.	Page 6
Risk of bias in systematic reviews, primary studies, and supplemental primary studies	18a	Present assessments of risk of bias or methodological quality for each included systematic review.	Page 6
	18b	Present assessments (*collected* from systematic reviews or *assessed* anew) of the risk of bias of the primary studies included in the systematic reviews.	Table 1
	18c	Present assessments of the risk of bias of supplemental primary studies (if included).	Page 6
Summary or synthesis of results	19a	For all outcomes, summarize the evidence from the systematic reviews and supplemental primary studies (if included). If meta-analyses were done, present for each the summary estimate and its precision and measures of statistical heterogeneity. If comparing groups, describe the direction of the effect.	Table 3
	19b	If meta-analyses were done, present results of all investigations of possible causes of heterogeneity.	Page 6
	19c	If meta-analyses were done, present results of all sensitivity analyses conducted to assess the robustness of synthesized results.	Page 6
Reporting biases	20	Present assessments (*collected* from systematic reviews and/or *assessed* anew) of the risk of bias due to missing primary studies, analyses, or results in a summary or synthesis (arising from reporting biases at the levels of the systematic reviews, primary studies, and supplemental primary studies, if included) for each summary or synthesis assessed.	Page 6
Certainty of evidence	21	Present assessments (*collected* or *assessed* anew) of certainty (or confidence) in the body of evidence for each outcome.	Page 6-7
**DISCUSSION**			
Discussion	22a	Summarize the main findings, including any discrepancies in findings across the included systematic reviews and supplemental primary studies (if included).	Page 14
	22b	Provide a general interpretation of the results in the context of other evidence.	Page 15
	22c	Discuss any limitations of the evidence from systematic reviews, their primary studies, and supplemental primary studies (if included) included in the overview of reviews. Discuss any limitations of the overview of reviews methods used.	Page 15
	22d	Discuss implications for practice, policy, and future research (both systematic reviews and primary research). Consider the relevance of the findings to the end users of the overview of reviews, e.g., healthcare providers, policymakers, patients, among others.	Page 15
**OTHER INFORMATION**			
Registration and protocol	23a	Provide registration information for the overview of reviews, including register name and registration number, or state that the overview of reviews was not registered.	Page 3
	23b	Indicate where the overview of reviews protocol can be accessed, or state that a protocol was not prepared.	Page 3
	23c	Describe and explain any amendments to information provided at registration or in the protocol. Indicate the stage of the overview of reviews at which amendments were made.	N/A
Support	24	Describe sources of financial or non-financial support for the overview of reviews, and the role of the funders or sponsors in the overview of reviews.	Page 15
Competing interests	25	Declare any competing interests of the overview of reviews' authors.	Page 16
Author information	26a	Provide contact information for the corresponding author.	Page 1
	26b	Describe the contributions of individual authors and identify the guarantor of the overview of reviews.	Page 15
Availability of data and other materials	27	Report which of the following are available, where they can be found, and under which conditions they may be accessed: template data collection forms; data collected from included systematic reviews and supplemental primary studies; analytic code; any other materials used in the overview of reviews.	Page 16

SUPPLEMENTARY FILE S2. Search strategy in electronic databases.

**PUBMED. Date of Search: 02/09/2024. Number of results: 1.081**		
**#**	**Search string**	**# of results**
1	((meta analy*[tiab] or metanaly*[tiab] or metaanaly*[tiab]) OR ((systematic*[tiab] OR evidence*[tiab]) AND (review*[tiab] or overview*[tiab])) OR "Systematic Review" [Publication Type] OR "Meta-Analysis" [Publication Type]) NOT (protocol[ti] OR scoping[ti])	941,817
2	"social media"[MeSH Terms] OR "social media"[tiab] OR "social network"[tiab] OR "social networking"[MeSH Terms] OR "social network*"[tiab] OR "communications media"[MeSH Terms] OR "communication media"[tiab] OR "communications media"[tiab] OR ((virtual[tiab] OR online[tiab] OR social[tiab]) AND (communit*[tiab] or network*[tiab] OR media[tiab])) OR Facebook[tiab] OR Twitter[tiab] OR Instagram[tiab] OR Snapchat[tiab] OR tiktok[tiab] OR YouTube[tiab] OR Pinterest[tiab] OR Linkedin[tiab] OR Tumblr[tiab] OR MySpace[tiab]	614,666
3	"educational intervention"[tiab] OR "health education"[MeSH Terms] OR (health[tiab] AND (education[tiab] OR literacy[tiab])) OR "health education"[tiab] OR "health literacy"[tiab] OR "health literacy"[MeSH Terms]	516,466
	"health personnel"[MeSH Terms] OR "health personnel"[tiab] OR "healthcare professional"[tiab] OR "healthcare provider"[tiab] OR ((health[tiab] OR healthcare[tiab]) AND (professional[tiab] OR professionals[tiab] OR provider[tiab] OR providers[tiab] OR personnel[tiab]))	998,492
4	#1 AND #2 AND #3 AND #4	1,123
5	Limit: 2001- to date	1081
**Web of Science. Date of Search: 02/09/2024. Number of results: 660**		
**#**	**Search string**	**# of results**
1	TS=((“meta-analysis” OR metanaly* OR metaanaly* OR "Systematic Review") OR ((systematic* OR evidence*) AND (review* OR overview*)) NOT (protocol OR scoping))	1,267,957
2	TS=("social media" OR "social network" OR "communication media" OR "communications media" OR ((virtual OR online OR social) AND (communit* or network* OR media)) OR Facebook OR Twitter OR Instagram OR Snapchat OR tiktok OR YouTube OR Pinterest OR Linkedin OR Tumblr OR MySpace)	878,966
3	TS=("health personnel" OR "healthcare professional" OR "healthcare provider" OR ((health OR healthcare) AND (professional OR professionals OR provider OR providers OR personnel)))	468,367
4	TS=(“educational intervention” OR (health AND (education OR literacy)) OR "health education" OR "health literacy")	379,769
5	#1 AND #2 AND #3 AND #4 AND PY=(2001-2024)	660
**CINAHL & PsycInfo. Date of Search: 02/09/2024. Number of results: 392**		
**#**	**Search string**	**# of results**
1	TI ((“meta-analysis” OR metanaly* OR metaanaly* OR "Systematic Review") OR ((systematic* OR evidence*) AND (review* OR overview*)) NOT (protocol OR scoping)) OR AB ((“meta-analysis” OR metanaly* OR metaanaly* OR "Systematic Review") OR ((systematic* OR evidence*) AND (review* OR overview*)) NOT (protocol OR scoping))	502,589
2	TI ("social media" OR "social network" OR "communication media" OR "communications media" OR ((virtual OR online OR social) AND (communit* or network* OR media)) OR Facebook OR Twitter OR Instagram OR Snapchat OR tiktok OR YouTube OR Pinterest OR Linkedin OR Tumblr OR MySpace) OR AB ("social media" OR "social network" OR "communication media" OR "communications media" OR ((virtual OR online OR social) AND (communit* or network* OR media)) OR Facebook OR Twitter OR Instagram OR Snapchat OR tiktok OR YouTube OR Pinterest OR Linkedin OR Tumblr OR MySpace)	320,367

3	TI ("health personnel" OR "healthcare professional" OR "healthcare provider" OR ((health OR healthcare) AND (professional OR professionals OR provider OR providers OR personnel))) OR AB ("health personnel" OR "healthcare professional" OR "healthcare provider" OR ((health OR healthcare) AND (professional OR professionals OR provider OR providers OR personnel)))	407,065
4	TI (“educational intervention” OR (health AND (education OR literacy)) OR "health education" OR "health literacy") OR AB (“educational intervention” OR (health AND (education OR literacy)) OR "health education" OR "health literacy")	247,721
5	S1 AND S2 AND S3 AND S4 AND Publication years 2001-2024	392
**COCHRANE. Date of Search: 02/09/2024. Number of results: 58**		
**#**	**Search string**	**# of results**
1	MeSH descriptor: [Social Media] explode all trees	597
2	MeSH descriptor: [Social Networking] explode all trees	239
3	MeSH descriptor: [Communications Media] explode all trees	19,586
4	("social media" OR "social network" OR "communication media" OR "communications media" OR ((virtual OR online OR social) AND (communit* or network* OR media)) OR Facebook OR Twitter OR Instagram OR Snapchat OR tiktok OR YouTube OR Pinterest OR Linkedin OR Tumblr OR MySpace):ti,ab,kw	22,229
5	MeSH descriptor: [Health Educators] explode all trees	34
6	MeSH descriptor: [Health Literacy] explode all trees	725
7	(“educational intervention” OR (health AND (education OR literacy)) OR "health education" OR "health literacy"):ti,ab,kw	58,513
8	MeSH descriptor: [Health Personnel] explode all trees	15,720
9	("health personnel" OR "healthcare professional" OR "healthcare provider" OR ((health OR healthcare) AND (professional OR professionals OR provider OR providers OR personnel))):ti,ab,kw	45,074
10	#1 OR #2 OR #3 OR #4	40,332
11	#5 OR #6 OR #7	58,523
12	#8 OR #9	55,190
13	#10 AND #11 AND #12	2,436
14	#10 AND #11 AND #12 with Cochrane Library publication date Between Jan 2001 and Dec 2024, in Cochrane Reviews	58

SUPPLEMENTARY FILE S3. Excluded reviews.

	Reference and year	Main reason for exclusion
1	Osman et al., 2022	Out of scope
2	Pascual-vallejo et al., 2022	Out of scope
3	Melgaard et al., 2022	Out of scope
4	Pretorius et al., 2022	Out of scope
5	Venancio et al., 2022	Out of scope
6	Panahi et al., 2021	No acces
7	Feroz et al., 2021	Out of scope
8	Riccardi et al., 2021	Out of scope
9	Lee et al., 2021	Out of scope
10	Steves et al., 2021	Out of scope
11	Suman et al., 2021	Out of scope
12	Soloe et al., 2021	Out of scope
13	Cruz-Oliver et al., 2020	Out of scope
14	Martinengo. et al., 2020	Out of scope
15	Mahmood et al., 2020	Out of scope
16	Zarnowiecki 2020	Out of scope
17	Torloni et al., 2020	Out of scope
18	Seddighil et al., 2020	Out of scope
19	Scerbe et al., 2019	Out of scope
20	Scott et al., 2019	Out of scope
21	Brusamento et al., 2019	Out of scope
22	Dunleavy et al., 2019	Out of scope
23	Oikonomidi et al., 2019	Out of scope
24	Bervell et al., 2019	Out of scope
25	Bruguera et al., 2019	Out of scope
26	Pollock et al., 2019	Out of scope
27	Rouleau et al., 2019	Out of scope
28	Chan et al., 2018	Out of scope
29	Hasyim et al., 2018	Out of scope
30	Sa et al., 2018	Out of scope
31	Penny et al., 2018	Out of scope
32	Ploeg et al., 2018	Out of scope
33	Waller et al., 2017	Out of scope
34	Covolo et al., 2017	Out of scope
35	Taroco et al., 2017	Out of scope
36	Henry et al., 2017	Out of scope
37	Wang et al., 2017	Out of scope
38	Edwards et al., 2016	Out of scope
39	River et al., 2016	Out of scope
40	Fernandez-Luque et al., 2016	Out of scope
41	Guraya et al., 2016	Out of scope
42	Smailhodzic et al., 2016	Out of scope
43	Benetoli et al., 2015	Out of scope
44	Roberts et al., 2015	Out of scope
45	Hu et al., 2015	Out of scope
46	Chi et al., 2015	Out of scope
47	McAlpine et al., 2015	Out of scope
48	Benetoli et al., 2015	Out of scope

49	Tamony et al., 2015	Out of scope
50	Hamine et al., 2015	Out of scope
51	Batt-Rawden et al., 2014	Out of scope
52	Davies et al., 2014	Out of scope
53	Huijpers et al., 2013	Out of scope
54	Yu et al., 2012	Out of scope
55	Martin et al., 2011	Out of scope
56	Martin et al., 2011	Out of scope
57	Or et al., 2009	Out of scope
58	Chivu et al., 2008	Out of scope
59	Guo et al., 2007	Out of scope
